# Unfavourably altered plasma clot properties in patients with primary Raynaud’s phenomenon: association with venous thromboembolism

**DOI:** 10.1007/s11239-019-01805-0

**Published:** 2019-01-25

**Authors:** Joanna Żuk, Agnieszka Snarska-Drygalska, Krzysztof Piotr Malinowski, Elżbieta Papuga-Szela, Joanna Natorska, Anetta Undas

**Affiliations:** 1 Second Department of Internal Medicine, Jagiellonian University Medical College, Krakow, Poland; 2 Specialist Dermatological Clinic, Krakow, Poland; 3 Faculty of Health Science, Institute of Public Health, Jagiellonian University Medical College, Krakow, Poland; 4 John Paul II Hospital, Krakow, Poland; 5 Institute of Cardiology, Jagiellonian University Medical College, 80 Pradnicka St., 31-202 Krakow, Poland; 6 Faculty of Medicine and Health Sciences, Jan Kochanowski University, Kielce, Poland

**Keywords:** Fibrin, Fibrinolysis, Venous thromboembolism, Raynaud phenomenon

## Abstract

Associations of Raynaud’s phenomenon (RP) with venous thromboembolism (VTE) are unclear. We investigated the occurrence of RP together with prothrombotic state markers and fibrin clot properties in VTE patients. In this prospective cohort study we enrolled 360 patients free of known autoimmune disease. D-dimer, von Willebrand factor (vWF), plasma clot permeability (K_s_), clot lysis time (CLT) along with fibrinolysis activators and inhibitors were determined at least 3 months since the VTE event. The presence/absence of RP was diagnosed at least 6 months before VTE. Primary RP occurred in 57 subjects (17%) with a 3.6-fold higher prevalence among women. Patients with RP had 11% higher fibrinogen, 16% higher vWF, 5% lower K_s_, and 10% longer CLT (all p < 0.05). Females with RP (21%) had 6.6% lower K_s_, 11.2% longer CLT, and 18.5% higher vWF (all p < 0.05) compared with men. CLT was predicted by PAI-1 and vWF levels. Regression analysis showed that RP was a predictor of prolonged CLT in the whole patient group (OR 3.46, 95% CI 1.92–6.24) and in women following VTE (OR 2.75, 95% CI 1.31–5.78). Primary RP patients tend to form denser plasma fibrin clots displaying impaired lysability and increased endothelial damage. RP might be a novel risk factor for VTE, especially in women.

**Electronic supplementary material:**

The online version of this article (10.1007/s11239-019-01805-0) contains supplementary material, which is available to authorized users.

## Highlights

- Primary Raynaud’s phenomenon (RP) occurs relatively frequently among patients with venous thromboembolism (VTE).
- Primary RP is associated with formation of denser fibrin clots less susceptible to lysis.
- Primary RP is associated with increased endothelial damage.
- RP might be a novel risk factor for VTE, especially in women.

## Introduction

Raynaud’s phenomenon (RP) is a vasospastic disorder usually involving peripheral small vessels of the fingers or toes in response to cold and/or emotional stress. This disorder is characterized by an episodic pallor, followed by cyanosis because of slow blood flow, and then rubor reflects the reactive hyperaemic phase. Ischemia, de-oxygenation and hyperaemia are the sequence of a typical attack [1–4].

A prevalence of RP is estimated at 3–5% of the Caucasian general population and it may be even higher in cold climates living populations [5]. RP is fourfold more common in women and more prevalent among individuals aged from 20 to 60 years [6].

RP is categorized as primary (80% of patients) or secondary. The aetiology of primary RP is unclear, while secondary RP is associated with numerous condition [4]. RP is observed commonly in patients with systemic sclerosis (SSc), in particular those with calcinosis, Raynaud’s phenomenon, oesophageal dysmobility, sclerodactyly, telangiectasia (CREST), however RP occurs frequently also in patients with mixed connective tissue disease, systemic lupus erythematosus, poly- or dermatomyositis, and other systemic autoimmune diseases [6]. RP is also observed in the thoracic outlet syndrome, small- and medium-sized vessel vasculitis, polycythaemia, cryofibrynogenaemia, cold agglutinin disease, paraproteinaemia, vibration injury, and finally it can be provoked by drugs and toxins [1–4].

A number of haemostatic alterations have been reported in patients with RP, however mainly in those with its secondary form [2, 7, 8]. It has been suggested that endothelial cell activation and/or thrombosis in the digits could be involved in the pathogenesis of RP [1, 7]. Several reports demonstrated that blood viscosity, along with plasma concentrations of fibrinogen and gammaglobulins, were increased in RP subjects compared with healthy controls [8, 9]. Increased platelet activation has been reported in both primary and secondary RP associated with SSc [10]. Despite evidence for enhanced activation of the blood coagulation, to our knowledge, there have been no clinical studies demonstrating that RP is linked to venous thromboembolism (VTE).

Stable fibrin clot formation is the final step of blood coagulation in vivo. It is known that fibrin clots composed of compact networks are less susceptible to lysis [11]. Such properties are characteristic for the so-called prothrombotic fibrin clot phenotype, usually driven by increased fibrinogen and thrombin formation, and have been identified in patients with coronary heart disease [12–15], ischemic stroke [16], and VTE [17, 18]. Furthermore, patients with rheumatoid arthritis, antiphospholipid syndrome and eosinophilic granulomatosis with polyangiitis have also been found to display reduced plasma clot permeability and lysability [19–21]. No data on fibrin clot properties in subjects with RP have been published yet.

VTE is a common condition and its incidence rises with age [22]. The RP is not viewed as a risk factor for VTE. Therefore we evaluated the prevalence of RP among young and middle-aged VTE patients and tested the hypothesis that in such patients, similarly to other prothrombotic conditions, this coexistence is associated with more pronounced abnormalities in the structure and function of a plasma fibrin network.

## Materials and methods

### Patients

In this prospective cohort study we recruited 360 consecutive patients aged from 18 to 65 years with a history of deep vein thrombosis (DVT) or/and pulmonary embolism (PE) referred to the Center for Coagulation Disorders in Krakow, Poland, from June 2013 to March 2016.

Eligible patients had documented VTE treated with oral anticoagulants or low-molecular weight heparin for at least 3 months prior to the enrolment. Exclusion criteria were signs of acute infection or known connective tissue disease, severe inherited thrombophilia, known malignancy, recent ischemic stroke or myocardial ischemia, chronic kidney disease stage III–V, liver injury, peripheral arterial disease, and haematological diseases known to be associated with RP.

The diagnosis of DVT and PE was established as described in the Electronic Supplementary Material. Demographic and clinical data were collected by a questionnaire. Raynaud’s phenomenon was established at least 6 months before VTE based on clinical features and nailfold capillaroscopy findings [23]. The study was approved by the Commission on Bioethics of the Regional Board of Medical Doctors in Krakow (No. 135/KBL/OIL/2013), and written informed consent was obtained from all participants in accordance with the Declaration of Helsinki.

### Laboratory investigations

Blood samples were drawn from an antecubital vein with minimal stasis. Detailed laboratory technics are described in the Electronic Supplementary Material.

Thrombophilia, including protein C, protein S, or antithrombin deficiency, factor V Leiden and prothrombin mutation G20210A, was tested as described [20]. Antinuclear antibodies (ANA) were identified using an immunofluorescence method (Euroimmun, Lubeck, Germany). Positive results were defined by the presence of ANA at the 1:160 serum dilution [24].

Serum levels of anticardiolipin antibodies (aCL) and anti-β2-glycoprotein I antibodies (aβ2GPI) were measured by INOVA kits (San Diego, CA, USA). The aCL IgG ≥ 15 GPL and IgM ≥ 12.5 MPL were assumed as positive based on the cut-off values (the 99th percentile) established in healthy volunteers. Positive values for IgG and IgM aβ2GPI were ≥ 20 SGU and SMU, respectively [25, 26]. Lupus anticoagulant was determined using two coagulation assays as recommended [27].

### Fibrin permeation

Fibrin clot permeation was determined using a pressure-driven system [11, 12]. Briefly, 20 mmol/L calcium chloride and 1 U/mL human thrombin (Sigma, St Louis, MO, USA) were added to 120 µL citrated plasma. A permeation coefficient (K_s_), which indicates the pore size, was calculated from the equation: K_s_= Q × L × η/t × A × Δp, where Q is the flow rate in time t; L, the length of a fibrin gel; η, the viscosity of liquid (in poise).

### Lysis assays

Clot lysis time (CLT) was measured as described previously [28]. Briefly, citrated plasma was mixed with 15 mmol/L calcium chloride, 10,000-diluted human tissue factor (Innovin, Siemens) with a final concentration of 0.6 pM, 12 µmol/L phospholipid vesicles and 60 ng/mL recombinant tissue plasminogen activator (Boehringer Ingelheim, Ingelheim, Germany). The turbidity was measured at 405 nm at 37 °C. CLT was defined as the time from the midpoint of the clear-to-maximum-turbid transition to the midpoint of the maximum-turbid-to-clear transition.

### Statistical analysis

The study was powered to have a 90% chance of detecting a 10% difference in CLT using a p-value of 0.05, based on the values of CLT as described previously [28]. In order to demonstrate such a difference or greater, 23 patients were required in each group.

Data are expressed as mean and standard deviation (SD) or as median (interquartile range, IQR) as appropriate. The Shapiro–Wilk test was used to assess conformity with a normal distribution. The continuous variables between two groups were compared using Student’s t-test and Mann–Whitney U-test as appropriate. Categorical variables were analysed using Chi^2^ or Fisher’s exact test as appropriate. The optimal cut-off points for fibrin clot parameters were evaluated by receiver operator characteristic curves (ROC). Pearson correlation coefficients were determined to assess associations between variables. Multiple linear regression analysis (the forward stepwise method) was used to determine predictors of clot variables. To evaluate independent predictors of the lowest K_s_ < 6 × 10^−9^ cm^2^ and the longest CLT > 113 min, those values were grouped into quartiles based on the distribution in the whole population. These predictors were determined by multivariate logistic regression. Results were presented as odds ratios (ORs) with 95% confidence interval (CI) also after adjustment for fibrinogen. A two-sided p-value < 0.05 was considered statistically significant. Statistical analyses were performed with SPSS Statistic software and JMP®.

## Results

### Patient characteristics

As shown in Table A1, in the whole cohort the prevalence of RP was 17.5%. Positive aPL were found in 19 (5%) of VTE patients. After excluding patients with positive aPL, the final analysis included 341 patients, among them 57 subjects with RP (17%). Positive ANA, mainly at a titre 1:160, though without clinical signs of autoimmune diseases, were observed in 141 patients, including 40 (28%) subjects diagnosed with RP. As expected, the prevalence of RP was 3.6-fold higher in women than in men.

Patients with RP did not differ from the remainder with regard to co-morbidities, the type of VTE (provoked/unprovoked) or medications except for hypercholesterolemia that was less common in the former group (Table A1). Most of the routine laboratory parameters, including hsCRP, were similar in the two groups, but we observed 11% higher fibrinogen and 16% higher vWF antigen in patients with RP (Table A2).

When subjects with positive ANA were excluded, we found that in the ANA negative patients (n = 219), RP was associated with 29% higher plasma fibrinogen, 19% higher vWF and 15% higher platelet count. Serum hsCRP was doubled in the ANA negative patients with RP compared to those without RP (Table A3).

Comparative analysis of fibrinolysis activators and inhibitors for the whole group showed that TAFI, plasminogen, antiplasmin and PAI-1 levels were similar in patients with RP and those free of this phenomenon (Table A2). This also held true for RP patients with negative ANA (data not shown).

### Fibrin clot properties

As shown in Table A2, patients with RP had 5% lower K_s_ and 10% longer CLT compared with the remainder, indicating the formation of denser plasma fibrin clots displaying impaired clot lysability. These differences remained significant after adjustment for fibrinogen (both p < 0.01). The separate analysis for women only (n = 195, 57.2%) showed that those with RP (n = 42, 12.3%) were characterized by 6.6% lower K_s_, 11.2% longer CLT, and 18.5% higher vWF (all p < 0.05, Table A4). In the ANA negative subjects, RP was also associated with less favourable clot variables, as reflected by 8% lower K_s_, 14% longer CLT and 16% higher vWF (Table A3).

**Fig. 1:**
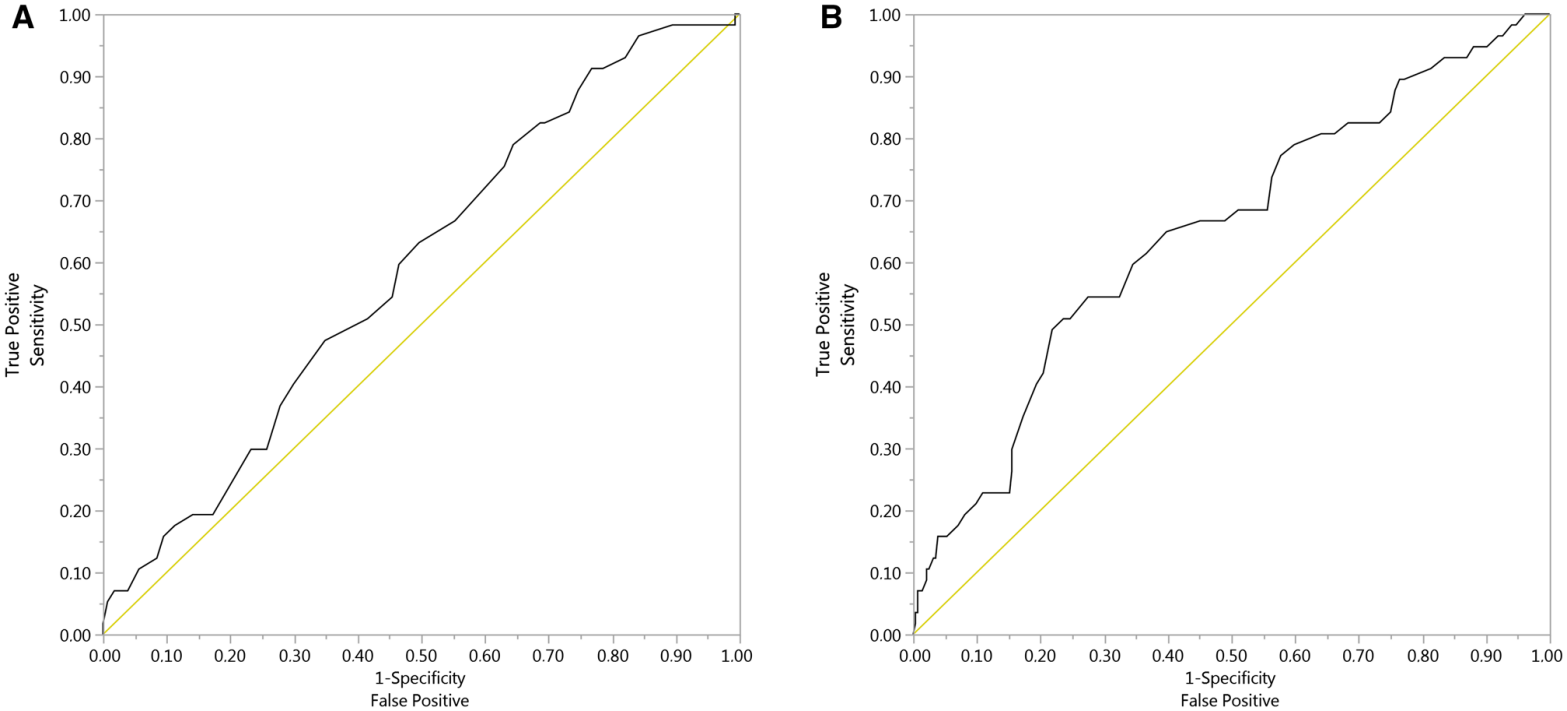
Receiver operating characteristic (ROC) curves for K_s_ (**a**) and CLT (**b**). **a** Using RAYNAUD = '1′ to be the positive level, AUC 0.59, 95% CI 0.51–0.67; **b** using RAYNAUD = ‘1’ to be the positive level, AUC 0.65, 95% CI 0.57–0.73

There were no associations of clot properties with demographics or clinical variables in the whole group. In subjects with RP (n = 57) fibrinogen and hsCRP were inversely associated with K_s_ (r = − 0.45, p = 0.0004, and r = − 0.42, p = 0.0012, respectively). A global fibrinolysis measure, CLT showed positive associations with PAI-1 (r = 0.33, p = 0.012) and vWF (r = 0.35, p = 0.0072), but not with TAFI, plasminogen or antiplasmin. Receiver operator characteristic analysis (Fig. 1) showed good accuracy for K_s_ and CLT to predict RP with the area under the curve (AUC) of 0.59 (95% CI 0.51–0.67) and 0.65 (95% CI 0.57–0.73), respectively. To demonstrate the independent effect of continuous variables on fibrin clot properties, a multiple regression model with adjustment for fibrinogen was used. In the whole patient group, PAI-1 and HDL-C were independent predictors of K_s_, while the independent predictors of CLT were PAI-1 and vWF (Table 1).

**Table 1:** Independent predictors of fibrin clot properties

Variable	Estimate (95% confidence interval)	p-Value
Predictors of K_s_		
PAI-1, ng/mL	− 0.03 (− 0.05; − 0.007)	0.01
HDL-C, mmol/L	0.19 (0.004; 0.38)	0.04
Predictors of CLT		
PAI-1, ng/mL	1.03 (1.01; 1.37)	< 0.001
vWF, %	0.10 (0.08; 0.14)	< 0.001

The logistic regression analysis adjusted for fibrinogen showed that prolonged CLT, defined as the top quartile, CLT > 113 min, but not low K_s_ was independently predicted by RP in both the whole patients group as well as this with negative ANA (OR 3.46, 95% CI 1.92–6.24, p < 0.001 and OR 5.89, 95% CI 2.32–14.94, p = 0.0002, respectively). Similar analysis for women only identified RP as an independent predictor of CLT (OR 2.75, 95% CI 1.31–5.78, p = 0.0075). Moreover, hsCRP was an independent predictor of both the longest CLT and the lowest K_s_, regardless of the ANA status (Table 2). Prolonged CLT was also independently predicted by vWF in both ANA positive and ANA negative groups (Table 2).

**Table 2:** Independent predictors of the lowest K_s_ and the longest CLT

Variable	Lower 95%	Upper 95%	p-Value
Predictors of K_s_			
Q1 K_s_ ANA positive (n = 341)			
hsCRP	1.05	1.20	0.0008
Q1 K_s_ ANA negative			
hsCRP	1.06	1.27	0.0016
Predictors of CLT			
Q4 CLT ANA positive			
hsCRP	0.85	0.98	0.046
vWF antigen	1.01	1.02	< 0.0001
Q4 CLT ANA negative			
hsCRP	1.00	1.18	0.041
vWF antigen	1.01	1.02	< 0.0001

## Discussion

This study is the first to demonstrate that primary RP is relatively common among young and middle-aged VTE patients, particularly among women, and this phenomenon is characterized by unfavourably altered plasma fibrin clot properties. We showed that plasma clots of patients suffering from primary RP display smaller pores and are degraded at a slower rate. Thus, the prothrombotic plasma clot phenotype may contribute to the pathogenesis of primary RP and increase the risk of thrombosis. Primary RP is a novel condition in which unfavourable fibrin alterations were demonstrated and they have been shown to be potent enough to be detectable following the VTE episode, which might per se enhance blood coagulation. It remains to be established whether similar clot characteristics can be demonstrated in primary RP prior to thrombotic events.

To our knowledge, this study is the first to evaluate associations of primary RP with thrombosis in patients who experienced provoked or unprovoked VTE. The prevalence of RP in our study performed in subjects aged 65 years or less was relatively high when compared to the most population-based surveys in which the estimated prevalence of RP is 3–5% [1]. This indicates that primary RP cases are overrepresented among VTE patients. In the literature, there is evidence linking RP with cardiovascular diseases, including arterial thromboembolic events, as demonstrated by Garner at al. in a systematic review of observational studies on primary RP [29]. An association of primary RP and cardiovascular diseases, including cardiovascular mortality, has also been reported [30, 31]. Nietert et al. suggested that RP might be a sign or precursor of undiagnosed vascular disease [31]. It might be speculated that RP is a risk factor for VTE of particular value among young and middle-aged women. However, a larger study on patients with primary RP is needed to validate this intriguing concept.

Clots more resistant to lysis with reduced permeability have already been described in several diseases associated with increased risk of arterial and/or venous thromboembolic events [13–20]. Our novel finding is that the coexistence of RP and VTE renders fibrin clots more prothrombotic, namely denser and more resistant to lysis compared with clot features observed in patients without this disorder. Although the differences are small, they are statistically significant also after adjustment for fibrinogen, a major determinant of fibrin clot features. Recent data have reported similar alterations in some systemic autoimmune conditions [19–21]. However, we found the impact of primary RP also when patients with positive ANA status were ruled out. The current study indicates that primary RP is an additional disorder associated with prothrombotic clot alterations. It might be hypothesized that prothrombotic mechanisms observed in VTE patients which are more pronounced in those with coexisting RP, might predispose to recurrent thrombotic events in this subset of patients.

Determinants of the prothrombotic clot phenotype observed in patients with RP are likely multiple and in part similar to other conditions [32]. It is to some extent driven by inflammatory changes associated with increased fibrinogen and CRP observed in the current RP patients. It has been demonstrated that CRP binds to fibrinogen and is associated with the formation of denser and more resistant to lysis fibrin clots [33]. We observed similar inverse associations of clot permeability with CRP concentrations, as reported in cardiovascular patients [13], which supports contribution of elevated CRP to plasma clot features. Moreover, the current study provides additional evidence for a role of an inflammatory state in unfavourable fibrin properties, reaching beyond the effect of elevated fibrinogen, since the differences between groups in fibrin clot characteristics remained significant after adjustment for this confounder.

Importantly, we found increased levels of circulating vWF in VTE patients with RP, suggesting an important contribution of endothelial cells activation and/or injury in this disease. Most published reports showed normal vWF plasma levels in patients with primary RP, while in subjects with secondary RP, these concentrations were significantly higher [34]. Elevated vWF has been shown to be associated with the generation of monocyte-and endothelial cell-derived microparticles, which might negatively affect clot features and modulate inflammation, blood coagulation and vascular function [35, 36]. It is also possible that increased vWF in patients with primary RP could identify the subjects at higher risk of developing an autoimmune systemic disease. In a recent observational study on 82 patients with RP, Gualtierotti et al. demonstrated higher levels of endothelial damage markers, including vWF, in subjects who subsequently developed a connective tissue disease, suggesting that vWF could predict such a progression towards a systemic disease [37]. This speculation needs a large-scale study with long-term follow-up.

The study has several limitations. First, the sample size was relatively limited, however, analyses were sufficiently powered and it is unlikely that the differences reported here result from a significant recruitment bias. Second, it was a cross-sectional study and our analysis was based on a determination of each variable at a single time point, therefore some changes in fibrin characteristics over time could be observed. Moreover, assessment of the same parameters prior to the VTE episode in a cohort of subjects with primary RP is needed to elucidate a role of altered fibrin features in this disorder. Prospective studies are required to assess the impact of RP on recurrent VTE, which might confirm its relevance as a new VTE risk factor. Our findings cannot be likely extrapolated after the first episode. Finally, we did not evaluate thrombin generation in this study, however previous reports failed to demonstrate increased thrombin formation in RP [38].

## Conclusions

This study shows that patients with primary RP have the prothrombotic clot phenotype at least in part associated with enhanced endothelial damage and inflammatory state. It might be speculated that the presence of RP may predispose to thromboembolic episodes given a high prevalence of this vascular disorder among VTE patients, particularly among young and middle-aged women. Further studies are required to elucidate clinical relevance of altered fibrin clot properties in the context of thrombotic risk among patients with various forms of RP.

## Electronic supplementary material

Below is the link to the electronic supplementary material.

Supplementary material 1 (DOCX 37 KB)
