# Supplementary material for: Unfavourably altered plasma clot properties in patients with primary Raynaud’s phenomenon: association with venous thromboembolism
Source: J Thromb Thrombolysis. 2019 Jan 25;47(2):248–54. doi: 10.1007/s11239-019-01805-0 (PMC6394442; doi:10.1007/s11239-019-01805-0)
Supplement: Supplementary file 1 — Supplementary material 1 (DOCX 37 KB) [file 11239_2019_1805_MOESM1_ESM.docx]

**Materials and methods**

***Patients***

The diagnosis of DVT was established by a positive finding on colour duplex sonography, while PE was diagnosed based on a positive finding on spiral-computed tomography. Unprovoked VTE was deﬁned as having no history of cancer, surgery, major trauma, plaster cast, hospitalization within 3 past months, pregnancy or delivery in the past 3 months.

***Laboratory investigations***

At the time of blood drawing none of the subjects was taking vitamin K antagonists (VKA). Patients on VKA were switched to enoxaparin for two weeks (at therapeutic doses) and blood was collected 18-24 h after the last injection (anti-Xa activity below 0.2 IU/ml) when International Normalized Ratio (INR) was <1.2. Lipid profiles, complete blood count, glucose, creatinine, homocysteine and INR were assayed by routine laboratory techniques. Fibrinogen was determined using the Clauss assay. High-sensitivity CRP (hsCRP) was measured by an immune turbidimetric assay (Siemens, Marburg, Germany). Immunoenzymatic assays were used to determine plasma D-dimer (Siemens), and plasminogen activator inhibitor-1 (PAI-1) antigen (American Diagnostica, Stamford, CT, USA). Plasma α2-antiplasmin and plasminogen were measured by chromogenic assays (Diagnostica Stago, Asniéres, France). Activated thrombin-activatable fibrinolysis inhibitor (TAFI) was measured by a chromogenic assay (American Diagnostica, Stamford, CT, USA). Plasma vWF antigen was measured by latex immunoassay on a STAR coagulation instrument (Diagnostica Stago, Asnieres, France).

***Fibrin clot analysis***

To evaluate fibrin clot properties, venous blood samples (3.2% trisodium citrate) were centrifuged at 2000×g for 10 min within 30 minutes of the draw, and the supernatant was aliquoted and stored at −80°C until analysis. All measurements were performed by technicians blinded to the origin of the samples. Intra-assay and inter-assay coefficients of variation were 5-7%.

**Table A1.** Patients characteristics.

| **Variable** | **Total (n=360)** | | | **APS negative (n=341)** | | |
| --- | --- | --- | --- | --- | --- | --- |
|  | **Raynaud patients**  **(n=63)** | **No Raynaud patients**  **(n=297)** | **P-value** | **Raynaud patients**  **(n=57)** | **No Raynaud patients (n=284)** | **P-value** |
| Age, years | 41.42±11.80 | 42.89±12.29 | 0.37 | 42.44±12.60 | 41.41±11.84 | 0.56 |
| Male, n (%) | 15 (24) | 134 (45.1) | 0.02 | 15 (26.3) | 131 (46.1) | 0.006 |
| BMI, kg/m^2^ | 27.38±6.29 | 27.25±5.24 | 0.69 | 27.44±6.31 | 27.24±5.32 | 0.75 |
| Current smoker, n (%) | 16 (25.40) | 61 (20.54) | 0.39 | 16 (28.07) | 59 (20.77) | 0.22 |
| Unprovoked VTE, n (%) | 32 (50.79) | 168 (56.57) | 0.40 | 28 (49.12) | 162 (57.04) | 0.27 |
| Trauma/surgery, n (%) | 9 (14.29) | 36 (12.12) | 0.52 | 7 (12.28) | 35 (12.32) | 0.42 |
| Pregnancy/postpartum*, n (%) | 4 (6.35) | 24 (8.08) | 0.36 | 4 (7.02) | 22 (7.75) | 0.40 |
| Contraceptives, HRT*, n (%) | 14 (22.22) | 54 (18.18) | 0.40 | 14 (24.56) | 51 (17.96) | 0.36 |
| Family history of VTE, n (%) | 25 (39.68) | 87 (29.29) | 0.11 | 23 (40.35) | 85 (29.93) | 0.12 |
| **Comorbidities**  Hypertension, n (%) | 21 (33.33) | 82 (27.61) | 0.36 | 17 (29.82) | 77 (27.11) | 0.68 |
| Diabetes, n (%) | 4 (6.35) | 13 (4.38) | 0.51 | 4 (7.02) | 12 (4.23) | 0.32 |
| Coronary heart disease, n (%) | 3 (4.76) | 5 (1.68) | 0.15 | 3 (5.26) | 5 (1.76) | 0.13 |
| Hypercholesterolemia, n (%) | 25 (39.68) | 178 (59.93) | 0.003 | 24 (42.11) | 170 (59.86) | 0.01 |
| Hypertriglyceridemia, n (%) | 12 (19.05) | 50 (16.84 | 0.67 | 12 (21.05) | 49 (17.25) | 0.49 |
| Hypothyreosis, n (%) | 8 (12.70) | 24 (8.08) | 0.24 | 7 (12.28) | 21 (7.39) | 0.29 |
| Asthma, n (%) | 2 (3.17) | 22 (7.41) | 0.28 | 1 (1.75) | 21 (7.39 | 0.14 |
| Superficial thrombosis, n (%) | 8 (12.70) | 24 (8.08) | 0.242 | 5 (8.77) | 24 (8.45) | 1.00 |
| Autoimmune disease, n (%) | 11 (17.46) | 9 (3.03) | <0.001 | 8 (14.04) | 7 (2.46) | 0.001 |
| Thrombophilia, n (%)** | 85 (28.62) | 22 (35.48) | 0.28 | ND | ND |  |
| **Treatment** |  |  |  |  |  |  |
| Beta-blocker, n (%) | 12 (19.05) | 65 (21.89) | 0.62 | 11 (19.30) | 59 (20.77) | 0.80 |
| ACE inhibitors, n (%) | 11 (17.46) | 45 (15.15) | 0.65 | 8 (14.04) | 39 (13.73) | 0.92 |
| Diuretics, n (%) | 5 (7.94) | 21 (7.07) | 0.79 | 5 (8.77) | 20 (7.04) | 0.58 |
| Statin, n (%) | 7 (11.11) | 37 (12.46) | 0.77 | 7 (12.28) | 34 (11.97) | 0.95 |
| Calcium blocker, n (%) | 5 (7.94) | 16 (5.39) | 0.39 | 5 (8.77) | 14 (4.93) | 0.34 |
| ASA, n (%) | 5 (7.94) | 17 (5.72) | 0.56 | 5 (8.77 | 17 (5.99) | 0.39 |
| Rivaroxaban, n (%) | 31 (49.21 | 136 (45.79) | 0.62 | 27 (47.37) | 129 (45.42) | 0.79 |
| VKA, n (%) | 18 (28.57) | 94 (31.65) | 0.63 |  |  |  |
| LMWH, n (%) | 8 (12.70) | 37 (12.46) | 0.89 | 8 (14.04) | 36 (12.67) | 0.85 |
| Results are expressed as number (percentage) or mean ±SD, VTE - venous thromboembolism, COPD – chronic obstructive pulmonary disease, HRT- hormonal replacement therapy, ACE- angiotensin converting enzyme, ASA – acetylsalicylic acid, VKA- vitamin K antagonist, LMWH – Low Molecular Weight Heparin, * Females only, **Factor V Leiden, prothrombin 20210 mutation or Protein S, Protein C, antithrombin deficiencies, ND – not determined | | | | | | |

**Table A2.** Results of laboratory tests in patients with Raynaud phenomenon (RP) compared with the remainder.

| **Variable** | **Total (n=360)** | | | **APS negative (n=341)** | | |
| --- | --- | --- | --- | --- | --- | --- |
|  | **Raynaud patients**  **(n=63)** | **No Raynaud patients**  **(n=297)** | **P-value** | **Raynaud patients**  **(n=57)** | **No Raynaud patients (n=284)** | **P-value** |
| **Routine parameters** | | | | | | |
| INR | 1.04±0.10 | 1.04±0.11 | 0.90 | 1.03±0.10 | 1.04±0.11 | 0.62 |
| aPTT, s | 27.64±3.81 | 27.73±3.7 | 0.81 | 27.44±3.95 | 27.71±3.73 | 0.52 |
| D-Dimer, ng/ml | 264 (180-377) | 211 (171-349) | 0.07 | 264 (182-373.5) | 209 (171-346.7) | 0.05 |
| Fibrinogen, g/l | 3.49±0.87 | 3.15 ±0.70 | 0.005 | 3.50±0.89 | 3.14±0.69 | 0.006 |
| Platelet count,10^3^/μl | 258.22±63.45 | 244.72 ±62.36 | 0.17 | 262±65.17 | 243.93±60.85 | 0.07 |
| tHcy, μmol/l | 11.6 ±3.9 | 11.8 ±4.9 | 0.95 | 11.5±4.04 | 11.9±4.9 | 0.75 |
| vWF, IU/ml | 204.43 ±56.07 | 174.13 ±49.22 | <0.001 | 203.44±54.47 | 174.95±49.15 | 0.0002 |
| hsCRP mg/l | 3.67 ±4.92 | 2.54 ±3.38 | 0.09 | 3.77±5.13 | 2.43±3.27 | 0.09 |
| **Fibrinolytic parameters** | | | | | | |
| PAI-1, ng/ml | 13.92 ±4.06 | 13.06 ±4.37 | 0.14 | 13.78±4.03 | 13.08±4.42 | 0.26 |
| Plasminogen, % | 105.32 ±16.72 | 104.67 ±17.21 | 0.45 | 105.16±17.31 | 104.63±17.17 | 0.55 |
| Antiplasmin, % | 102.14 ±14.1 | 104.38 ±15.95 | 0.30 | 102.14±13.49 | 102.24±15.74 | 0.35 |
| TAFI antigen, % | 103.89 ±21.66 | 106.56 ±20.99 | 0.11 | 103.89±22.32 | 106.43±21.07 | 0.11 |
| **Clot characteristics** | | | | | | |
| K_s_,10^-9^ cm^2^ | 6.54 ±0.85 | 6.89 ±0.96 | 0.013 | 6.55±0.86 | 6.87±0.96 | 0.03 |
| CLT, min | 104.94±17.18 | 95.34 ±16.02 | <0.001 | 104.56±17.81 | 95.50±15.88 | 0.0004 |
| Results are expressed as mean ±SD or median (interquartile range), INR – international normalized ratio, aPTT- activated partial thromboplastin time, D-D – D-Dimer level in clot lysis assay, Fbg – Fibrinogen, PLT – platelet count, hsCRP– high sensitivity C-reactive protein, tHcy – total homocysteine, TAFI – thrombin activatable fibrinolysis inhibitor; PAI-1– plasminogen activator inhibitor-1, K_s_ – permeability coefficient, CLT – clot lysis time | | | | | | |

**Table A3.** Laboratory investigations in patients with Raynaud phenomenon (RP) versus remainder according to the antinuclear autoantibody (ANA) status.

| **Variable** | **Total (n=360)** | | | **ANA negative (n=219)** | | |
| --- | --- | --- | --- | --- | --- | --- |
|  | **Patients with RP (n=63)** | **Patients without RP**  **(n=297)** | **P-value** | **Patients with RP**  **(n=23)** | **Patients without RP (n=196)** | **P-value** |
| INR | 1.04±0.10 | 1.04±0.11 | 0.90 | 1.04 ±0.13 | 1.04 ±0.11 | 0.588 |
| aPTT, s | 27.64±3.81 | 27.73±3.7 | 0.81 | 27.32 ±4.11 | 27.61 ±3.39 | 0.703 |
| D-Dimer, ng/ml | 264 (180-377) | 211 (171-349) | 0.07 | 258 (181-370) | 201 (171-337) | 0.090 |
| Fibrinogen, g/l | 3.49±0.87 | 3.15 ±0.70 | 0.005 | 3.97 ±0.98 | 3.08 ±0.7 | <0.0001 |
| Platelets, 10^3^/µl | 258.22±63.45 | 244.72 ±62.36 | 0.17 | 280.13 ±72.07 | 243.99±60.07 | 0.02 |
| hsCRP, mg/l | 3.67 ±4.92 | 2.54 ±3.38 | 0.09 | 4.65 ±5.12 | 2.26 ±3.25 | 0.006 |
| tHcy, μmol/l | 11.63 ±3.91 | 11.88 ±4.86 | 0.95 | 10.96 ±3.33 | 11.96 ±4.96 | 0.58 |
| vWF,% | 204.43 ±56.07 | 174.13 ±49.22 | <0.001 | 209.39 ±56.59 | 175.64 ±49.36 | 0.002 |
| PAI-1, ng/ml | 13.92 ±4.06 | 13.06 ±4.37 | 0.14 | 14.03 ±4.39 | 13.33 ±4.29 | 0.46 |
| Plasminogen,% | 105.32 ±16.72 | 104.67 ±17.21 | 0.45 | 103.39 ±11.40 | 103.81 ±17.77 | 0.93 |
| Antiplasmin, % | 102.14 ±14.10 | 104.38 ±15.95 | 0.30 | 103.65 ±12.06 | 103.11 ±15.47 | 0.87 |
| TAFI antigen, % | 103.89 ±21.66 | 106.56 ±20.99 | 0.11 | 105.81 ±18.84 | 106.39 ±21.38 | 0.14 |
| K_s_, 10^-9^ cm^2^ | 6.54 ±0.85 | 6.89 ±0.96 | 0.013 | 6.29 ±0.74 | 6.83±0.92 | 0.013 |
| CLT, min | 104.94±17.18 | 95.34 ±16.02 | <0.001 | 110.43 ±20.92 | 96.55 ±16.44 | <0.0001 |
| Results are expressed as mean ±SD or median (interquartile range). Abbreviations see table 2. | | | | | | |

**Table A4.** Clinical characteristics and laboratory investigations in females with or without Raynaud phenomenon (RP).

| **Variable** | **APS negative female patients (n=195)** | | |
| --- | --- | --- | --- |
|  | **Females with**  **RP**  **(n=42)** | **Females without**  **RP**  **(n=153)** | **P-value** |
| Age, years | 42.55±12.57 | 39.19±11.00 | 0.11 |
| BMI kg/m^2^ | 25.5 (21.91-31.44) | 24.30 (21.79-29.32) | 0.28 |
| Current smoker, n (%) | 11(26.19) | 26 (16.99) | 0.17 |
| Unprovoked VTE, n (%) | 19 (45.24) | 63 (41.18) | 0.63 |
| Family history of VTE, n (%) | 16 (38.10) | 49 (32.03) | 0.45 |
| **Comorbidities** |  |  |  |
| Hypertension, n (%) | 13 (30.95) | 33 (21.57) | 0.20 |
| Diabetes, n (%) | 4 (9.52) | 7 (4.58) | 0.25 |
| Coronary heart disease, n (%) | 2 (4.76) | 1 (0.65) | 0.11 |
| Hypercholesterolaemia, n (%) | 17 (40.48) | 82 (53.59) | 0.13 |
| Hypertriglicerydaemia, n (%) | 5 (11.90) | 16 (10.46) | 0.78 |
| Hypothyreosis, n (%) | 1 (2.38) | 3 (1.96) | 0.66 |
| Asthma, n (%) | 1 (2.38) | 7 (4.58) | 1.00 |
| Superficial thrombosis, n (%) | 4 (9.52) | 11 (7.19) | 0.74 |
| Autoimmune disease, n (%) | 5 (11.90) | 3 (1.96) | 0.012 |
| Inherited Thrombophilia, n(%) | 13 (31.71) | 39 (25.49) | 0.42 |
| **Treatment** |  |  |  |
| Beta-blocker, n (%) | 10 (23.81) | 35 (22.88) | 0.89 |
| Angiotensin converting enzyme inhibitors, n (%) | 4 (9.52) | 15 (9.80) | 1.00 |
| Diuretics, n (%) | 3 (7.14) | 10 (6.54) | 1.00 |
| Statin, n (%) | 4 (9.52) | 17 (11.11) | 1.00 |
| Calcium-blocker, n (%) | 2 (4.76) | 5 (3.27) | 0.64 |
| Aspirin, n (%) | 3 (7.14) | 9 (5.88) | 0.72 |
| Rivaroxaban, n (%) | 19 (45.24) | 67 (43.79) | 0.86 |
| Vitamin K antagonist, n (%) | 11 (26.19) | 5 (33.33) | 0.74 |
| **Routine parameters** |  |  |  |
| International Normalized Ratio | 1.01 (0.98-1.07) | 1.02 (0.98-1.08) | 0.66 |
| Activated Partial Thromboplastin Time, s | 27.01±3.73 | 27.27±3.78 | 0.76 |
| D-Dimer, ng/ml | 265.5 (182.5-378.5) | 247.0 (171.0±401.0) | 0.64 |
| Fibrinogen, g/l | 3.66±0.91 | 3.18±0.65 | 0.002 |
| Platelet count,10^3^/µl | 265 (215-302) | 262 (212-295) | 0.57 |
| Total homocysteine, μmol/l | 9.90 (8.75-12.48) | 9.80 (7.60-12.10) | 0.033 |
| Von Willebrand Factor, % | 208.19±51.90 | 175.71±50.25 | 0.27 |
| **Fibrinolytic parameters** |  |  |  |
| Plasminogen activator inhibitor type 1, ng/ml | 13.2 (10.8-17.8) | 12.10 (9.70-16.60) | 0.23 |
| Plasminogen, % | 105.0 (95.8-114.5) | 103.00 (92.50-116.0) | 0.46 |
| Antiplasmin, % | 102.52±14.59 | 102.14±16.24 | 0.88 |
| TAFI antigen, % | 101.6 (91.3-109.8) | 105.4 (97.0-118.2) | 0.16 |
| **Clot characteristics** |  |  |  |
| K_s_,10^-9^ cm^2^ | 6.49±0.93 | 6.95±0.98 | 0.006 |
| CLT, min | 106.69±18.74 | 95.93±16.96 | 0.0005 |
| Results are expressed as number (percentage) or mean ±SD , for abbreviations see Tables A1, A2 | | | |

Abbreviations: VTE, venous thromboembolism; TAFI, thrombin activatable fibrinolysis inhibitor; K_s_, fibrin clot permeability coefficient, CLT, clot lysis time.
